# The hospital outpatient alcohol project (HOAP): protocol for an individually randomized, parallel-group superiority trial of electronic alcohol screening and brief intervention versus screening alone for unhealthy alcohol use

**DOI:** 10.1186/1940-0640-8-14

**Published:** 2013-09-03

**Authors:** Natalie A Johnson, Kypros Kypri, John B Saunders, Richard Saitz, John Attia, Adrian Dunlop, Christopher Doran, Patrick McElduff, Luke Wolfenden, Jim McCambridge

**Affiliations:** 1 School of Medicine and Public Health, University of Newcastle, Level 4 West, HMRI Building, University Drive, Newcastle, Callaghan NSW 2308, Australia; 2 Centre for Youth Substance Abuse Research, University of Queensland, Herston, Queensland, Australia; 3 Disciplines of Psychiatry and Addiction Medicine, University of Sydney, Sydney, NSW, Australia; 4 Clinical Addiction Research and Education Unit, Boston University Schools of Medicine and Public Health and Boston Medical Center, Boston, MA, USA; 5 Department of General Medicine, John Hunter Hospital, Newcastle, Australia; 6 Hunter Medical Research Institute, Newcastle, NSW, Australia; 7 Hunter New England Local Health District Drug and Alcohol Clinical Services, Newcastle, NSW, Australia; 8 Hunter New England Local Health District Population Health, Newcastle, NSW, Australia; 9 Faculty of Public Health & Policy, London School of Hygiene & Tropical Medicine, London, UK

**Keywords:** Alcohol, Screening, Brief intervention, Internet, Intervention, Clinical trials, Hospital outpatients

## Abstract

**Background:**

Electronic screening and brief intervention (e-SBI) is a promising alternative to screening and brief intervention by health-care providers, but its efficacy in the hospital outpatient setting, which serves a large proportion of the population, has not been established. The aim of this study is to estimate the effect of e-SBI in hospital outpatients with hazardous or harmful drinking.

**Methods/Design:**

This randomized controlled trial will be conducted in the outpatient department of a large tertiary referral hospital in Newcastle (population 540,000), Australia. Some 772 adults with appointments at a broad range of medical and surgical outpatient clinics who score 5–9 inclusive on the Alcohol Use Disorders Identification Test-Consumption (AUDIT-C) subscale will be randomly assigned in a 1:1 ratio to electronic alcohol screening alone (control) or to e-SBI. As randomization will be effected by computer, researchers and participants (who will be invited to participate in a study of alcohol use over time) will be blinded to group assignment. The primary analysis will be based on the intention-to-treat principle and compare weekly volume (grams of alcohol) and the full AUDIT score with a six-month reference period between the groups six months post randomization. Secondary outcomes, assessed six and 12 months after randomization, will include drinking frequency, typical occasion quantity, proportion who report binge drinking, proportion who report heavy drinking, and health-care utilization.

**Discussion:**

If e-SBI is efficacious in outpatient settings, it offers the prospect of systematically and sustainably reaching a large number of hazardous and harmful drinkers, many of whom do not otherwise seek or receive help.

**Trial registration:**

Australian New Zealand Clinical Trials Registry ACTRN12612000905864.

## Background

Unhealthy alcohol use is third in the list of leading risk factors for premature deaths and disability globally [1], and one in five Australian adults consume alcohol at a level that elevates their risk of alcohol-related disease or injury over their lifetime [2]. Alcohol screening and brief intervention has been shown to reduce unhealthy alcohol consumption in primary care patients who are not dependent on alcohol [3], and “brief advice for hazardous drinking”, which refers to a level or pattern of alcohol consumption that increases the risk of harmful consequences for the drinker and others, is included as a “good buy” in the World Health Organization’s list of interventions to tackle noncommunicable disease risk factors [4].

Although a number of national and international organizations recommend that alcohol screening and brief intervention be routinely implemented in a variety of health-care settings [4-7], it is underutilized [8]. In Australia, for example, counselling or advice in relation to alcohol is provided at a rate of about 0.4 per 100 encounters in the primary care setting [9]. Barriers to implementation by health-care providers include time constraints, concerns about patient sensitivity to questions about alcohol consumption, insufficient training in administering brief interventions, and absence of specific reimbursement for these services [10]. Electronic screening and brief intervention (e-SBI) is a viable alternative because it circumvents many of these provider-level barriers; however, the majority of randomized controlled trials testing the efficacy of e-SBI have been conducted with university students (mainly young people) who have high rates of binge drinking [11]. The efficacy of e-SBI for adults across the lifespan, who have a broad range of drinking patterns, has not been established.

Australian public hospitals provided 16.7 million specialist outpatient clinic services in 2010–2011 [12], creating an untapped opportunity to provide alcohol screening and brief intervention to a large number of users of the public health-care system. Since findings regarding the efficacy of alcohol screening and brief intervention for hospital outpatients are mixed [13-18], and, to our knowledge, no studies evaluating the efficacy of e-SBI for hospital outpatients have been published, this study aims to determine the efficacy of e-SBI for hospital outpatients who report hazardous or harmful drinking. The primary hypothesis is that the intervention group will consume 3.5 fewer standard drinks (35 g ethanol) relative to the control group six months after intervention.

## Methods/Design

### Design

**Figure 1:**
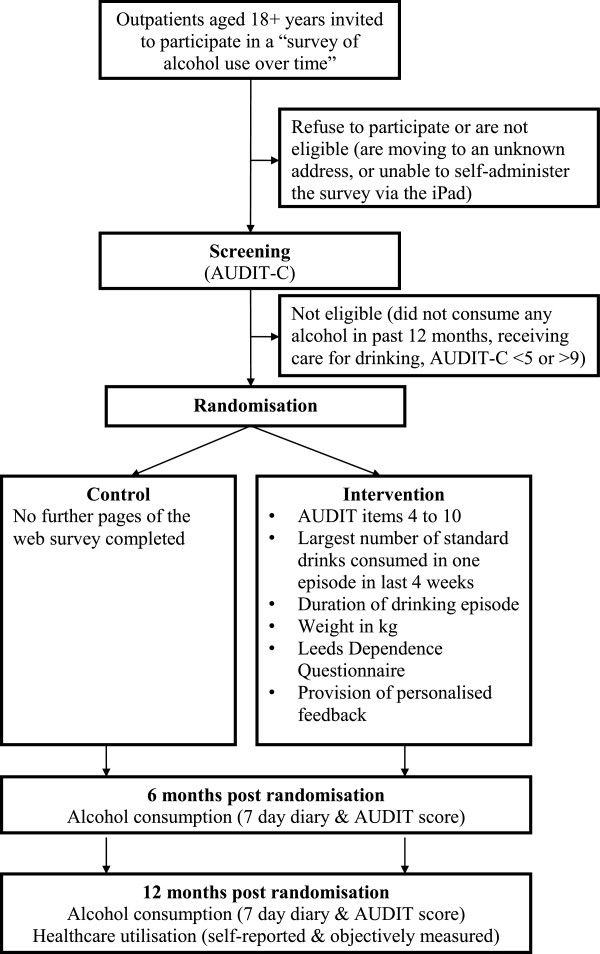
Trial design.

The study is a two-arm parallel group, individually randomized controlled trial designed to determine the superiority, or otherwise, of e-SBI (intervention condition) compared with screening alone (control condition) in reducing alcohol consumption in hospital outpatients who screen positive for hazardous or harmful drinking (Figure 1). Follow-up assessments will occur six and 12 months post-randomization. The trial will be reported in accordance with the Consolidated Standards of Reporting Trials (CONSORT) Statement [19]. Ethical approval has been obtained from the Hunter New England Human Research Ethics Committee (12/05/16/4.04) and the University of Newcastle Human Research Ethics Committee (H-2012-0272).

### Setting

The study will be conducted in the Ambulatory Care Center (outpatient department) at the John Hunter Hospital, a large tertiary referral hospital located in Newcastle, Australia (population 540,000) [20]. A broad range of medical and surgical outpatient services are provided by the Ambulatory Care Center, including rehabilitation; transplant; vascular access; vascular surgery; pain management; oral and maxillofacial surgery; colorectal care; ear, nose, and throat (ENT) and head and neck surgery; general surgery; neurosurgery; ophthalmology; orthopedics; and urology. Patients attending these clinics must have a written referral from their primary care provider and may be attending this hospital, even if it is not the closest to their home, because the widest range of services is generally provided by large public hospitals in Australia.

### Eligibility criteria

Outpatients 18 years of age or older who are capable of providing written informed consent, capable of self-administering the e-SBI using an iPad, and are not moving to an unknown address in the next 12 months (may become lost to follow-up) will be invited to participate. Consenting outpatients who have not consumed any alcohol in the past 12 months, who are currently receiving treatment for their drinking, or who screen positive for possible alcohol dependence will be excluded prior to randomization.

### Screening

Research staff will log eligible consenting outpatients into the e-SBI program on an iPad using a unique study number. Page 1 of the e-SBI will describe the *Hospital Outpatient Alcohol Project* (HOAP). Page 2 will collect demographic data (gender, age, and postcode) and contact information (email address). Page 3 will ask respondents if they have consumed alcohol in the last 12 months (yes/no). Those who respond “no” will be excluded at this point. Page 4 will ask respondents if they are receiving treatment for alcohol-related problems (yes/no). Those who respond “yes” will be excluded at this point. Page 5 will comprise the three alcohol consumption questions from the Alcohol Use Disorders Identification Test (AUDIT) [21]. These questions comprise the validated three-item screening tool known as the AUDIT-C [22], which has specificity and sensitivity similar to the full 10-item AUDIT. The AUDIT-C will be used to screen outpatients for hazardous and harmful alcohol consumption because completion of the full AUDIT has been shown to produce reductions in self-reported alcohol consumption [23]. Upon clicking the continue button on this page, AUDIT-C scores will be calculated (range 0–12 with higher scores reflecting more severe drinking problems). Participants who score less than 5 will be excluded at this point. A minimum score of 5 points was selected because it has high specificity while maintaining good sensitivity for identifying patients with hazardous or harmful drinking [22]. Participants who score more than 9 will also be excluded at this point because the probability of alcohol dependence with an AUDIT-C score above 9 is high [24], and these patients probably require more than brief intervention [25]. Participants in the control group will not be asked other questions about alcohol consumption at baseline, because answering questions on drinking in brief intervention trials appears to alter subsequent self-reported behavior [26].

### Allocation and blinding

Participants will be randomly assigned (simple randomization with 1:1 allocation, no blocking, no stratification) to one of two treatment groups: electronic alcohol screening alone (control group) or electronic alcohol screening plus further assessment and personalized feedback via an iPad (intervention group). Allocation concealment will be ensured as the random assignment will be computer-generated (SecureRandom.random_number method in Ruby [27]) and effected immediately following screening via a hand-held device (iPad). Participants will be blind to the true nature of the study, as they will be asked to consent to participate in a series of surveys on alcohol use among outpatients and will not be aware that they have been randomized. Data analysts will be blinded to group allocation when they prepare and clean the data. Only after the data has been locked will unblinding occur and the analysis be completed. No other blinding is required as there are no care providers (the intervention is delivered via iPads), and there are no outcome assessors (the study outcomes are self-reported by blinded participants).

### Intervention

The intervention will be informed by a decade of research by Kypri and colleagues [28-31] and will comprise two parts: (1) additional assessment using an iPad and (2) electronic personalized feedback, including normative feedback which has been shown to reduce alcohol consumption in heavy drinking university students [32] and adult problem drinkers [33].

The assessment questions will comprise (1) the remaining six questions of the AUDIT [21]; (2) a question regarding the largest number of standard drinks (in Australia, this refers to a drink containing 10 g alcohol) consumed on a single occasion in the last four weeks; (3) a question regarding the duration of the drinking episode in hours; (4) body weight (for the purpose of estimating peak blood alcohol concentration [BAC]); and (5) the Leeds Dependence Questionnaire (LDQ) [34].

The electronic personalized feedback will comprise (1) the participant’s AUDIT score and guidance on its meaning [21]; (2) an estimated BAC for their heaviest drinking episode in the previous month with information on the behavioral and physiological sequelae of their BAC, along with traffic-crash relative risk if they reported consuming alcohol in the past 4 weeks; (3) an estimate of their spending on alcohol in the past year; (4) a bar graph comparing their typical episodic consumption with medical recommendations [35] and that of adults of the same age and gender [36]; (5) a bar graph comparing weekly consumption with medical recommendations [35] and that of adults of the same age and gender [36]; and (6) an LDQ score with an explanation of the associated health risk [34]. It is important to note that normative feedback via the bar charts will be suppressed when participants’ episodic or weekly consumption is consistent with medical recommendations [35]. In addition to the electronic personalized feedback, three pages offering facts about alcohol (for example, the health consequences of unhealthy alcohol consumption), tips for reducing the risk of alcohol-related harm and sources of support for drinking problems (for example, contact details for services available in the local health district) will be included. The time to complete the intervention and read the electronic personalized feedback is expected to be less than 10 minutes [37]. A copy of the electronic personalized feedback will be emailed or posted to participants who agree to this information being sent to them.

### Outcomes

Although 79% of Australian households have access to the internet at home, home internet access is more common in households with higher incomes [38]. Therefore, outcomes will be assessed six months post-randomization by email, or by post in the case of participants who do not provide an email address. The co-primary outcomes are (i) weekly volume (number of standard drinks [grams of alcohol] consumed in the past week) and (ii) AUDIT score with a reference period of the past six months. The weekly volume will be assessed using a seven-day retrospective diary [39]. Participants will be asked to indicate how many standard drinks they consumed over the past seven days. For example, “Yesterday it was [insert name of the day], and I consumed [insert number] standard drinks.” Secondary outcomes that will be assessed six months post-randomization include (i) drinking frequency (number of drinking days in the past week); (ii) typical occasion quantity (number of standard drinks per drinking day in the past week); (iii) proportion who report binge drinking (i.e., exceed the recommended upper limit for risk of acute harm by consuming more than four standard drinks on any occasion in the past week [35]); and (iv) proportion who report heavy drinking (i.e., exceed the recommended upper limit for risk of chronic harm by consuming more than 14 standard drinks in the past week [35]). In contrast to the previous version [40], there is no gender difference in the current *Australian Guidelines to Reduce Health Risks from Drinking Alcohol*[35].

The secondary outcomes to be assessed 12 months post-randomization are: (i) weekly volume; (ii) AUDIT score with a reference period of the past six months; (iii) drinking frequency; (iv) typical occasion quantity; (v) the proportion who report binge drinking; (vi) the proportion who report heavy drinking; and (vii) health-care utilization (e.g., proportion who have visited the emergency department, proportion admitted to hospital, and proportion who utilize outpatient services if available).

### Follow-up and minimization of attrition

Since attrition reduces the effective sample size and can introduce bias, strategies shown to increase response to electronic and postal questionnaires will be employed [41,42]. Six and 12 months post-randomization, participants will be sent a letter reminding them about the study and advising them that they will receive a brief follow-up questionnaire in the next few days. A $20 supermarket voucher, delivered in advance and redeemable irrespective of further participation, will be enclosed with the letter as a token of appreciation. Supermarket vouchers have been used in trials conducted by the authors previously [28-30] and have been shown to improve response rates [42]. Participants who provide an email address receive an emailed hyperlink to a brief web-based follow-up questionnaire, while participants who do not provide an email address will receive a paper questionnaire. Up to three email/postal and SMS reminders will be sent following the initial invitation to complete the follow-up surveys. Participants who do not respond to the initial and reminder emails/postal surveys will be followed up via telephone.

### Data management

Baseline data will be collected using iPads. The data collected via the iPads will be transmitted to a password-protected server located in a secure area in the Ambulatory Care Center through a wireless router and downloaded into Microsoft Access (2010 version) daily. Research staff will log participants into the e-SBI program using a unique study number so that baseline and follow-up data can be linked. Follow-up data will be collected via a web-based follow-up questionnaire when participants provide an email address, and by post otherwise. Where follow-up data is collected electronically, the unique study number will be embedded in the hyperlink to the web-based follow-up questionnaire emailed to each participant [43]. Where follow-up data is collected by post, the unique study number will be printed on the questionnaire. Participants’ study information will be linked with health-care utilization data collected by the health service using their Medical Record Number (MRN), which is a unique patient identifier for public hospitals in the Hunter New England Local Health District. Double data entry in Microsoft Access (2010 version) will be undertaken for data collected by post.

### Sample size

The estimate relates to the first co-primary outcome and is based on findings from a pilot study conducted in the same setting in 2010. The mean number of standard drinks consumed by outpatients who participated in the pilot study was 20 (SD = 15). Assuming a 5% level of significance and 80% power, we would require 289 participants per group (578 total) at six months to detect a difference of 3.5 standard drinks (35 g ethanol) per week (18%) between the groups, as found by Kypri et al. [29] and similar to the 38 g ethanol reduction found in the meta-analysis by Kaner et al. [3]. Assuming 25% attrition at six months post-randomization, which is conservative given that attrition was 15% at 12 months in a study utilizing similar methods conducted by Kypri and colleagues [29], 772 participants will be randomized at baseline.

### Statistical methods

The first co-primary outcome (number of standard drinks consumed in the past week at six months post-randomization) and the secondary outcome, also related to number of standard drinks, will be analyzed with negative binomial regression. Similarly, number of inpatient, outpatient, and emergency-department presentations will be analyzed using negative binomial regression. The secondary outcome of number of drinking days in the past week will be analyzed using logistic regression within a generalized estimating equation framework to take into account the repeated measurements on individuals. Each individual’s data will be entered as the binomial outcome of yes/no to drinking on each day. The proportions meeting guidelines will also be analyzed using logistic regression. The AUDIT score will be analyzed using linear regression. All analyses will control for baseline AUDIT-C score [44] as in previous trials reported by the investigators [29]. Participants will be analyzed in the group to which they were randomized (intention-to-treat). Sensitivity analyses using pattern mixture models will be conducted to explore the potential impact of any missing follow-up data [45,46]. Additional analyses will be conducted to determine whether the intervention was more efficacious among (1) patients with a lower AUDIT-C score; (2) patients in the intervention group who requested that a copy of their electronic personalized feedback be sent to them; (3) younger people; (4) men; and (5) patients who reside in a relatively advantaged postal area [47].

## Discussion

The Hospital Outpatient Alcohol Project is a two-arm parallel group randomized controlled trial designed to generate an estimate of the efficacy of e-SBI in a setting that provides health care to a large number of users of the public health-care system who are not seeking treatment for their drinking. If e-SBI is efficacious, analyses to determine its cost-effectiveness will be undertaken.

Strengths of the study include the use of a proven recruitment procedure, a valid screening instrument, recent Australian normative data, allocation concealment from research staff, the blinding of participants, and the inclusion of health-care utilization as an objective outcome measure. The main limitation is the use of self-report for the co-primary outcome measures. Although blood markers (for example, gamma-glutamyltransferase level) may seem preferable for assessing outcomes, they are insufficiently sensitive to hazardous drinking, and self-report has generally been found to be reliable [48,49], particularly via computers [50,51].

To our knowledge, this will be the first trial of the efficacy of e-SBI in a large group of general hospital outpatients. Although e-SBI will not, by itself, eliminate hazardous and harmful drinking in hospital outpatients because of environmental influences on this behavior, producing evidence on the efficacy of an infinitely scalable intervention with high fidelity will assist health services to provide preventive interventions to individuals as recommended by the World Health Organization [4], and Australian organizations such as the Royal Australian College of General Practitioners [6] and the Preventative Health Taskforce [52].

## Competing interests

The authors declare they have no competing interests.

## Authors’ contributions

All authors contributed to the design of the protocol. NJ and KK produced the initial draft of the manuscript; all authors read, provided editorial input, and approved the final manuscript.
